# Dementia and Cognitive Impairment in the Australian Farming Community. A Preliminary Qualitative Investigation of Healthcare Provider's Perceptions of Risks/Barriers to Care

**DOI:** 10.1111/ajr.70006

**Published:** 2025-02-22

**Authors:** James Gunning, Kerri‐Lynn Peachey, Tony Lower, Carlos Mesa‐Castrillon

**Affiliations:** ^1^ School of Rural Health, Faculty of Medicine and Health The University of Sydney, Orange Campus Orange New South Wales Australia

## Abstract

**Background:**

Farmers are at higher risk of developing dementia due to occupational exposures throughout their lives. People living in regional and remote areas also have increased barriers to care compared with urban populations, while farmers face additional barriers.

**Aims:**

To explore the barriers to care and risks faced by farmers with dementia, from the perspectives of healthcare workers. This preliminary study also aims to explore the differences in barriers faced by farmers and non‐farming rural populations.

**Methods:**

Seven participants from a range of health professions were interviewed in a semi‐structured style to explore their experiences. The data were coded and analysed using a constructivist grounded theory approach to search for recurring themes.

**Results:**

Six key themes emerged from the data: (1) barriers arising from typical farmer personality traits; (2) geographic isolation; (3) late diagnoses of dementia; (4) barriers to the provision of care on the farm; (5) on‐farm risks; and (6) transition to residential aged care home.

**Conclusions:**

Farmers living with dementia on‐farm may face significant barriers to care and risks to themselves and others compared to non‐farm rural and urban populations. These additional barriers predominantly stem from increased geographic isolation and personality traits common to farming populations. However, there is potential for change to improve care provision through earlier diagnosis, more efficient service funding to enhance treatment availability and the use of residential aged care homes more suited to farmers.

**Summary**

- What is already known on this subject?
  ○ Farming populations face additional barriers to accessing care compared to rural populations. There are also perceived barriers specific to rural populations diagnosed with dementia. These include the stigma of the disease leading to a reluctance to ask for help, a lack of confidence in General Practitioners (GPs) to diagnose and manage the disease, plus a reluctance to ask for help from others in the community who have their health burdens to manage.
- What this paper adds?
  ○ Healthcare providers identified considerable barriers that farmers with dementia may face. These barriers stem from an interplay between inherent personality qualities common to many farmers and geographical factors. These factors act as barriers to both the timely diagnosis of the disease and the ability to provide effective care for these populations in the community.

## 1 Introduction

It is currently estimated that there are over 400 000 people in Australia living with dementia, with current demographic trends expecting this to increase to over 850 000 by 2050 [1]. While certain risk factors for developing dementia are well studied, including age, gender, genetics, physical activity/inactivity, smoking, alcohol and weight [2], there is limited and conflicting evidence on the effects of environmental risk factors on developing the disease. There are links between some pesticides and dementia risk [3] especially with exposure to the neurotoxic pesticide groups, organophosphates and organochlorides [4, 5]. These risk factors point to a possible explanation for the 46% increased risk of developing dementia in populations with a long history of agricultural work [6].

Populations residing in regional and rural areas have increased barriers to accessing dementia‐related care compared with urban populations. These include structural barriers such as reduced access to specialist care [7, 8, 9], high turnover of healthcare providers [10, 11], increased travel distances for services [8] and higher financial burdens compared with urban populations [8, 9]. There are also a number of perceived barriers specific to rural populations diagnosed with dementia, including the stigma of the disease leading to a reluctance to ask for help [8, 10], a lack of confidence in GPs to diagnose and manage the disease [7, 8], and a reluctance to ask for help from others in the community who have their own health burdens to manage [10]. These barriers may explain the increased healthcare avoidance rates in rural populations, which are 1.7 times higher than in urban populations [12]. In addition, due to the limited provision of healthcare for dementia in rural and remote Australia, people with dementia, their families and carers have restricted services regarding diagnosis, education about their condition, and limited access to a range of quality dementia support and services [13]. Dementia Australia, the national peak body for dementia reported a shortage of healthcare providers and poor access to health services, as well as socioeconomic disadvantage, which limits their ability to access specialists [13].

In assessing the literature, no study could be identified that studies the barriers to care faced by farmers with dementia. However, literature exploring the barriers to care for farmers with haematological malignancies [14] and mental health concerns [15], showed that similar barriers exist between farming and non‐farming rural populations. Notwithstanding this, farming populations faced additional barriers. These include an inability to leave the farm to access care due to their need to work, difficulties in finding or paying for a manager to run the farm in their absence, plus a determination to maintain ownership of the property for generational succession in the face of financial pressures arising from healthcare needs [14, 15]. It has also been found that farmers, in general, showed a lack of concern over minor or early symptoms of disease [15]. This has culminated in the finding that farmers were found to be half as likely to visit a GP compared with non‐farm workers living in rural areas [16].

The objective of this study is to (1) explore the perceptions of healthcare workers regarding both the barriers to care experienced and the risks for farmers with dementia living on the farm, and (2) to compare these barriers and risks with non‐farmers living in the community in a rural setting.

## 2 Methods

This preliminary qualitative study sought healthcare professionals who had experience caring for farmers with dementia in community settings throughout Australia. These participants were purposively recruited through existing professional connections at regional hospitals (Orange and Dubbo), alongside social media advertising. The research team included three members of AgHealth Australia (TL, KLP and CMC) with previous experience in qualitative research, a Research Professor from the University of Sydney (DL), and a third‐year medical student (JG) who received workshop training in the principles of qualitative research. Potential participants were screened via email to determine if they met the inclusion criteria (over 18 years of age, current or retired healthcare worker, experience caring for farmers with dementia in a community setting, reside in Australia). Eligible participants signed a consent form and subsequently were interviewed between June and July 2024. There were no prior relationships between the interviewer and participants. The interview questions were designed to explore participant's experiences of caring for farmers while living on the farm, from diagnosis to the decision to move a farmer from their property and into a residential aged care home. The list of questions was determined from the aims of this research project and was validated through consensus with the research team (Appendix S1). Interviews took the form of a guided conversation and the participants received instructions to skip a question or to stop the interview and withdraw immediately in case of feeling distressed. A male member of the research team (JG) conducted individual semi‐structured interviews over the phone (according to the participant's choice), which were audio recorded and transcribed using Microsoft Word's transcription function. A member of the research team (JG) analysed and coded the transcripts using nVivo 14 software to search for recurring themes. A constructivist grounded theory approach was used in the analysis with a combination of inductive and deductive thematic analysis [17]. The output of the first two interviews was analysed and discussed in consultation with the research team. This allowed for iterative changes to the questions asked in subsequent interviews to further explore the key themes emerging. Transcripts were offered to the participants.

COREQ guidelines for qualitative studies guided the report (Appendix S2) [18]. Ethics approval to conduct the study was granted by the Greater Western Human Research Ethics Committee 2024/ETH00231. Participants gave their consent prior to participation.

## 3 Results

Semi‐structured interviews of between 18‐ and 24‐min duration, with an average length of 21 min (SD 2.6 min) were conducted with seven healthcare workers (two geriatricians, three nurses, one occupational therapist and one physiotherapist). Their experience in working with farmers with dementia ranged from 6 to 25 years. These healthcare workers had worked in the NSW, WA, NT, ACT, QLD and VIC. There were no follow‐up interviews with any participants. The participants' demographic information is shown in Table 1.

**TABLE 1:** Participant's demographic information.

Characteristic	*n* (%)
Gender	
Male	2 (29)
Female	5 (71)
Profession	
Geriatrician	2 (29)
Nurse	3 (43)
Physiotherapist	1 (14)
Occupational therapist	1 (14)
State	
New South Wales	5 (71)
Western Australia	3 (43)
Northern Territory	1 (14)
Australian capital territory	1 (14)
Queensland	1 (14)
Victoria	1 (14)

Interviews of the study participants' perspectives on the barriers to care and risks of farmers with dementia living on farm yielded five key themes: (1) typical farmer personality traits; (2) geographic isolation for farmers; (3) late diagnoses of dementia; (4) barriers to the provision of care on farm, (5) risks of remaining on farm and (6) transition to residential aged care home. There is also some overlap between the ideas presented, with some codes applying to multiple themes. The relationship between themes and codes is shown in Table 2.

**TABLE 2:** Major themes and their respective codes.

Theme	Codes
Typical farmer personality traits	Identity as a farmer Stoicism Fear of losing the way of life Healthcare avoidance Not asking for help Not wanting to leave the farm
Geographic isolation	Geographic isolation Social isolation Loneliness Lack of community support
Late diagnosis of dementia	Fear of raising concerns Hard to diagnosis Limited formal services Late diagnosis Incidental diagnosis Not recognising signs of disease Insight issues
Barriers to the provision of care on the farm	Acceptance of care Not accepting diagnosis Financial barriers Reliance on family Not asking for help Refusal of care Telehealth
Risks of remaining on the farm	Firearms Machinery Driving Misadventure Finances Livestock
Transition to residential aged care homes	Family members' decision Progression of dementia symptoms Increase of care requirements Safety of carers and farmers Agricultural aged care homes

### 3.1 Farmers' Personality Traits

Healthcare workers noted a common desire for independence and privacy, ‘farmers are incredibly proud people and quite reasonably private people, who mind their own business, get on with the job, and don't really like people making too much of a fuss’ (Participant 03, 50F). Healthcare workers noted that farmers in turn see a diagnosis of dementia as a threat to their ability to continue to live the lifestyle that they have developed, ‘so farmers in their essence, throughout their lives often need to work very independently in isolation, work things out themselves, very self‐sufficient, and so often a diagnosis of dementia is seen as a threat to that’ (Participant 04, 55F). This was mentioned as a reason for farmers to withhold retirement, as a great degree of purpose is derived from their identity as a farmer and a farming family:

One thing that sometimes motivates farmers to continue farming and living on the farm for much longer than people in other occupations would continue to work is a desire to keep the farm in the family… I've observed multiple farmers continuing to work into an advanced age really because they don't want to sell. (Participant 01, 40M)

Other healthcare workers noted a high degree of stoicism within farming populations ‘(there is) a personality type in amongst them and their family members to be very, very stoic, to crack on with what's practical’ (Participant 02, 33F). A combination of these factors was described to lead to an increased perception of denial and difficulty accepting the diagnosis of dementia, ‘I think a lot of them are in denial’ (Participant 05, 31M), ‘I have found in my experience, older men seem to have more difficulty accepting dementia diagnosis’ (Participant 04, 55F). These personality traits, accompanied by the insight problems associated with the disease, were noted to mask the symptoms of the disease in these populations, ‘they don't realise that they're not coping until somebody starts to pick up the pieces for them, and then they realise’ (Participant 03, 50F), leading to a degree of healthcare avoidance within the population, ‘the biggest barrier to care is actually asking for help in the first place’ (Participant 01, 40M).

### 3.2 Geographic Isolation for Farmers

Participants reported high degrees of geographic isolation for farmers diminishing the effectiveness of services, ‘when your home care provider can provide the, you know, 13 hours a fortnight of domestic assistance, but it takes 4 hours for that provider to get there, you only get 8 hours or 9 hours due to distance’ (Participant 03, 50F). There was a high level of concordance among participants about geographic isolation being a considerable barrier, ‘(it is) quite complicated actually because of people being able to access stuff on a farm, a lot of services just won't go out to rural or like remote communities or to farming properties to do things at their home’ (Participant 02, 33F). Similarly, ‘especially with rough terrain, getting a nurse physically to the farm is near impossible, it rains and 60kms of dirt roads becomes impossible’ (Participant 07, 26F). Furthermore, accessing in‐town services is also problematic, ‘the transports, because getting someone to medical appointments is such a big, like it's a whole day and a lot of these guys, they don't want to take a day off’ (Participant 05, 31M).

### 3.3 Late Diagnoses of Dementia

Participants also noted a clear pattern of late diagnosis within the population of farmers with dementia, ‘In my anecdotal experience is that farmers with dementia often have a formal diagnosis substantially later than non‐farmers’ (Participant 01, 40M), ‘there's certainly a degree of sort of, like I said, stoicism, before they would present with a diagnosis, so they're often a lot more advanced’ (Participant 02, 33F). This is combined with a perceived higher rate of incidental diagnoses than non‐farmers, ‘I think probably because farmers are less likely to seek healthcare for miscellaneous things, so I think it's probably caught later, and it's usually in response to like an accident or a fall’ (Participant 03, 50F). The healthcare workers expressed differing views on the reasons for late presentations, with some discussing stoic‐like qualities, ‘they tend to look after themselves, they choose to do things on their own, keep things private, and it's not until things are really bad that then they seek help’ (Participant 03, 50F). Others discussed structural barriers with lack of access to regular primary healthcare as reasons for late presentations and missed diagnoses, ‘So if you're not seeing a medical professional regularly… because the locum GP (has) come in for a few months and then they go, well they just don't pick up on these things so they don't follow up with stuff’ (Participant 05, 31M). It was also noted that the loss of insight that is associated with dementia could act as a reason for these later presentations, ‘There might be problems with insight in the patient, in the farmer with dementia that mean that they don't feel that there is problem which means, “Why would I go and see a doctor or a geriatrician about it?”’ (Participant 01, 40M).

### 3.4 Barriers to the Provision of Care on the Farm

Many of the participants also discussed how, even once a formal diagnosis of dementia was made, the barriers to their treatment persisted. These included the patient denying the diagnosis of dementia and refusing care, ‘if you're a farmer with dementia and you don't agree that you've got a problem and you don't think you need help, you're more likely to refuse that help when it's offered’ (Participant 01, 40M). Others noted inherent fears about losing their way of life and being forced off the farm and into assisted living residential aged care homes, ‘they don't wanna go, you know the “you're not taking me, you're not putting me in a home” sort of stuff. You know, there is that sort of mentality’ (Participant 03, 50F). While others discussed the paucity of care services that existed in rural and remote areas, ‘I think farmers, like people in country areas, have limited access anyway and then you add that to farmers who really are reluctant to engage with health services’ (Participant 06, 47F). There was strong concordance in the opinions of participants that these barriers to providing formal care have led to the families assuming the bulk of the care requirement, ‘I would say for those who do have supportive families, the families take on the greater burden of that care’ (Participant 03, 50F), ‘I'd say primarily it's family and depending on where they're living, if they're living in a more supportive town, then the town can be quite good’ (Participant 05, 31M), ‘because a lot of the (formal) care is very inflexible, it's often easier to just do it yourself and then become reliant on family members and like I was saying, like even employees to step up and fill those gaps’ (Participant 02, 33F).

### 3.5 Risks of Remaining on the Farm

The participants also discussed a number of key risks for farmers with dementia remaining on their farm, to both themselves and others around them. These included risks around driving, both on public and private roads, ‘So there are risks associated with driving when it's no longer safe to do so, either on private roads, or on private roads in the paddock or on public roads. I've had patients who've had major car accidents in that setting’ (Participant 01, 40M). Risks associated with the use and storage of firearms on properties:

So people living on farms are more likely to have firearms than those living in town, and if you've got dementia with BPSD (behaviours and psychological symptoms of dementia), that can be a risk just to patients themselves, or family members. (Participant 01, 40M)

Risks associated with machinery and tools, especially if the farmer with dementia is still actively working on the farm, ‘anything that you would be worried about Granddad in suburbia, times that by 10, because they have access to large machinery, larger tools’ (Participant 03, 50F). Risks associated with wandering, getting lost and general misadventure considering the large sizes of many properties, ‘it's probably that they're getting lost on the farm and being in trouble and not being able to seek help or people not knowing where they are to get help to them if they need it’ (Participant 04, 55F). It was also noted that farmers with dementia who were still actively farming posed risks to livestock through neglect, ‘there are risks to animals that they would be neglected, the patient with dementia who has poor insight can be essentially neglecting their livestock’ (Participant 01, 40M).

### 3.6 Transition to Residential Aged Care Home

Ultimately, family members may decide to move a farmer from their property and into a residential aged care home, when carers can no longer safely care for the farmer with dementia:

Where it gets quite tricky and difficult, is when someone would go from being on the farm straight into residential care, and that can often be triggered by perhaps the health of the of the primary carer, and the risks around, you know, the safety particularly if people are wandering and particularly at night if they are, you know outside and becoming disorientated. (Participant 04, 55F)

It was suggested residential aged care homes in rural areas that provide a similar agricultural lifestyle, ‘…if they can have like residential aged care where it was much more suited to like what a farmer's normal environment would be, that would make it a lot easier to for family to make that decision… that's where their loved one would be most well cared’. (Participant 02, 33F).

## 4 Discussion

Participants noted there were considerable barriers to care faced by farmers with dementia who remain living on farms. These barriers stem from an interplay between inherent personality qualities common to many farmers and geographical factors. These factors act as barriers to both the timely diagnosis of the disease and the ability to provide effective care for these populations in the community.

Key stoic personality characteristics that have been developed by farmers throughout their lives, and potentially due to their independent and work‐dominated lifestyles, act as a barrier to diagnosis. This is manifested as a prominent healthcare avoidance in farming populations [12] which ultimately leads to a later diagnosis of dementia compared with rural non‐farm and urban populations. This healthcare avoidance is exacerbated by the difficulty of accessing a regular GP in regional and rural Australia [19]. Both long wait times and a high degree of turnover are cited as reasons for why farmers with an inclination towards healthcare avoidance will delay raising concerns [15]. Participants also spoke of the practical reasons for travelling long distances, requiring time away from the farm and farm work to access healthcare as reasons for healthcare avoidance. These results are in line with previous research by Kelly and Yarwood (2018) on farmers and farming families living with dementia in the United Kingdom, which highlights that ‘Many farmers “do not want to make a fuss” and need to be persuaded to use them’ [20]. This study also suggested that farmers felt disadvantaged (in terms of access to care services), by living in a rural location and working on a farm, especially because of issues such as bad weather, poor roads and distance [20].

The social isolation associated with living away from town can also lead to later diagnoses of dementia. The lack of continued observation from neighbours and other community members means that small changes in cognition are less likely to be noticed and concerns less likely to be raised. Similarly, Kelly and Yarwood (2018) highlighted that people with dementia in rural areas are particularly at risk from loneliness, given that farms are further entangled with their simultaneous use as both a place of business and a place of home [20].

Other barriers to diagnosis are associated with the fear of what effect this diagnosis will have on their ability to retain their way of life. Farmers may express fears that this disease would ultimately force them off their property and into a residential aged care home. A direct result of this was a common finding of patients denying any changes to their cognition and a refusal to seek help. All participants expressed that many of these barriers to an early diagnosis of dementia were common to rural non‐farm populations, especially those due to a lack of access to regular primary healthcare. The additional barriers arising from inherent personality traits were, however, noted to be considerably more dominant in farming populations. McGrath (2015) also reported that farmers in Australia with haematological diseases face similar barriers to care such as financial hardship in accessing care and maintaining the farm in their absence, difficulty in travelling for care, carer absence in remote areas and dilemma to move out of the property for care [14].

Once a formal diagnosis of dementia is made in a farmer living on their farm, there are still considerable barriers to providing adequate care that will allow them to continue to live safely on their farm. The main reason for these barriers is the geographical isolation of these rural properties. Most participants cited this as the main barrier to providing care, as many formal services are unable to reach these properties, or if they can provide at‐home care, much of the funded hours of care are taken up through commuting to these areas. A direct result of the inflexible and insufficient provision of at‐home care for farmers with dementia is that the burden of care falls onto the family of the farmer. It was noted that while some families can provide this care, the majority report a high degree of carer stress [21]. It was also noted that the ultimate decision to move a farmer from their property and into a residential aged care home is mostly brought about when the family reaches a point where they no longer feel that they can safely care for the farmer with dementia. This is related to the progression of the disease and an increase in care requirements. However, this is another important consideration when attempting to keep the farmer living on the farm for as long as possible.

The barriers associated with providing adequate care were expressed to be common between farming and non‐farming populations in rural areas. It was noted that those populations living in rural or regional centres still faced barriers associated with a lack of health professional services available to them. Many participants noted a lack of service providers and a difficulty in hiring new healthcare professionals for rural areas, which acted as barriers to providing effective care to these populations, which is consistent with other findings [10, 11]. Workforce challenges found in this study are also in line with a government report that shows lower numbers of clinical Full‐Time Equivalent (FTE) provided by medical practitioners in remote and very remote areas compared to major cities [22].

Our results also are consistent with a literature review that aimed to summarise the impact of travel on cancer patients. The review reported that travel to treatment may be perceived as a barrier and is reported as inconvenient and a practical hardship [23]. Similarly, our results are in line with the Australian government report that highlights people living in very remote areas can face barriers to accessing and using health care. The additional time and transportation costs to access health care services may delay access to care [24]. Potential intervention strategies to enhance healthcare access for farmers with dementia might include increasing the provision of services to farmers through grants, public‐private partnerships, incentive programmes for healthcare professionals, telehealth expansion, community‐based funding and policy advocacy. For aged care homes, incorporating familiar farm elements, involving the community, forming local partnerships and implementing sustainable practices can help create a supportive environment that aligns with the agricultural lifestyle. Additionally, these strategies could also help alleviate current shortages in health workforce supply in rural and remote Australia [22].

The results of this study should be interpreted cautiously and may not be relevant to all rural areas across Australia. This is due to both the small sample size (as a preliminary assessment) and the self‐selection bias that arose from the recruitment strategy. There is also potential bias from the semi‐structured interview style as participants' responses were based on the questions asked, despite the best efforts from the interviewer to ask non‐leading questions. However, this study did recruit participants from a range of healthcare professions and from individuals that worked across numerous states/territories, which will reduce the potential for any systemic bias in the responses. Another limitation of this study is not reaching data saturation due to several reasons. (1) Low recruitment rates of health care providers that have worked with farmers with dementia, despite social media advertisements Australia‐wide. (2) Time constraints due to the limited recruitment duration period of 3 months related to the availability of the research team. Previous studies have acknowledged farmers are a hard‐to‐access population [20], therefore, it could be expected that limited numbers of healthcare providers have worked with this population. Not reaching data saturation implies our findings may not fully represent the range of perspectives or experiences relevant to healthcare providers' perspectives. Therefore, important themes or patterns might remain undiscovered, and the findings may be less generalizable to other contexts or populations. During the process of study consent participants had the option to request a review of their transcript; however, no participants requested to review their transcript. The lack of participant feedback might limit the credibility of the results.

This study raises opportunities for further research attempting to validate the perspectives of healthcare workers with a wider and more comprehensive assessment, particularly around late diagnoses and risk‐related injuries. Additionally, a key avenue for further investigation should be into the perspectives and experiences of carers on the barriers to care and risks to these populations. This may provide a different perspective on the challenges faced and be used to validate or contest the views of healthcare professionals, which ultimately would allow for a more holistic understanding of the issues to allow for a more effective provision of care to farmers living on farms. Additionally, further research should focus on innovative interventions to overcome the barriers farmers with dementia face in accessing healthcare including specialist shortages in remote Australia.

## 5 Conclusion

The key conclusion from this study is that there are areas to improve in providing better care for farmers with dementia and to allow them to continue living safely on the farm. These include promoting regular engagement with healthcare professionals to detect signs of disease earlier, providing more funding for the provision of services to farmers that consider the travel time required to reach the property and developing more residential aged care homes in rural areas that provide farmers with a way of life similar to the one they are familiar with. This would not only promote their quality of life but also may encourage them to move off the farm earlier in their disease progression, which can minimise the risks they face on their isolated properties.

## Author Contributions

**James Gunning:** methodology, data curation, investigation, formal analysis, project administration, writing – original draft, writing – review and editing. **Kerri‐Lynn Peachey:** conceptualization, methodology, investigation, writing – review and editing, supervision, writing – original draft. **Tony Lower:** conceptualization, methodology, investigation, supervision, writing – review and editing, writing – original draft. **Carlos Mesa‐Castrillon:** conceptualization, methodology, supervision, investigation, project administration, writing – original draft, writing – review and editing.

## Ethics Statement

Ethics approval to conduct the study was granted by the Greater Western Human Research Ethics Committee 2024/ETH00231.

## Conflicts of Interest

The authors declare no conflicts of interest.

## Supporting information


Appendix S1.

Appendix S2.

## Data Availability

The data that support the findings of this study are available on request from the corresponding author. The data are not publicly available due to privacy or ethical restrictions.
