# Supplementary material for: Dementia and Cognitive Impairment in the Australian Farming Community. A Preliminary Qualitative Investigation of Healthcare Provider's Perceptions of Risks/Barriers to Care
Source: Aust J Rural Health. 2025 Feb 22;33(1):e70006. doi: 10.1111/ajr.70006 (PMC11845917; doi:10.1111/ajr.70006)
Supplement: Supplementary file 1 — Appendix S1. [file AJR-33-0-s001.docx]

Appendix 1:

**Intro:**

Hello [participant’s_name],

My name is Anonymised and today I will be asking you some questions to understand your views on the risks and barriers to care for people with dementia and cognitive impairment. In particular, we will be looking at any variations between farmers that live on farm and individuals that reside in rural towns.

The interview could take up to 20 minutes. Please let me know if you need to stop at any point. We can take a break, or if you need, we can stop the interview altogether.

If you agree, I will start audio recording the interview and ask you some questions to make sure that you are okay to go ahead. Are you ready to start?

[Press record button]

I have started recording now.

Can you confirm that you have been provided with a Participant Consent Form?

Can you confirm that you have read the Participant Consent Form and agree to all the points included in the form?

Do you agree to provide your consent to take part in this research study?

Would you like to ask me any questions before we start?

**Demographic Questions:**

1. What is your age?
2. What is your gender?
3. What is your highest educational degree?
4. What is your profession?
5. How many years of experience providing healthcare for people with dementia do you have?
6. What states have you worked in?
7. Do you have experience caring for farmers with dementia?
8. How do you check the occupation of your patients?

**Specific questions:**

1. How are farmers typically diagnosed with dementia?
   1. How does this differ to rural populations?
2. In your experience, how do farmers respond to a diagnosis of dementia?
   1. How does this differ from other rural populations?
3. What is the typical care pathway following a formal diagnosis of dementia in regards to input from health services?
   1. Does this differ from other rural populations?
4. What are their typical support networks?
   1. Have you found farmers prefer to rely on informal or formal care?
5. In your opinion, what are the biggest barriers to care for farmers?
   1. How do these differ from other rural populations?
6. What are the risks of a farmer with dementia remaining on their farm?
   1. How do these differ from other rural populations?
7. What are some potential strategies to mitigate these risks?
8. What are some potential strategies to overcome these barriers to care?

**End:**

Thank you so much [participant’s_name]. Is there is anything you would like to add before we finish the interview?

That concludes the interview.

I am now stopping the recording.

Thank you so much for your time today. I really appreciate you taking the time to talk to me about your experiences.

*[End interview]*
